# Green Hydrothermal Synthesis of N-doped Carbon Dots from Biomass Highland Barley for the Detection of Hg^2+^

**DOI:** 10.3390/s19143169

**Published:** 2019-07-18

**Authors:** Yadian Xie, Dandan Cheng, Xingliang Liu, Aixia Han

**Affiliations:** 1 Chemical Engineering College, Qinghai University, Xining 810016, China; 2 School of Life Science, Wuchang University of Technology, Wuhan 430223, China

**Keywords:** N-doped carbon dots, hydrothermal method, biomass, highland barley, Hg^2+^ ion

## Abstract

Totally water-soluble N-doped Carbon dots (N-CDs) were synthesized by a green hydrothermal method from biomass using Highland barley as a carbon source and ethanediamine as nitrogen source. TEM and XRD showed the graphitic amorphous structure and narrow diameter distribution of these N-CDs. N-doping to the crystal lattice and carrying many hydrophilic groups on the surface of N-CDs were verified by XPS and FT-IR. The as-synthesized N-CDs emitted strong blue fluorescence at 480 nm and owned a relatively high quantum yield of 14.4%. The product also could sensitively and selectively detect Hg^2+^ ions in the range of 10–160 μM and the limit of detection was equal to 0.48 μM.

## 1. Introduction

Carbon dots (CDs) have attracted increasing attention due to their unique characteristics such as excellent optical and electronic properties, good water-solubility, low toxicity, and perfect biocompatibility [1,2,3,4,5,6] among others. A number of synthetic methods have been used to produce CDs, for example arc-discharge [7], laser ablation [8], pyrolysis [9], electro-oxidation [10], and hydrothermal treatment [11] etc. Every technique has its own distinctive feature, and therefore obtains diverse CDs. Among these, nitrogen-doped CDs (N-CDs), owing to their predominant performance in fluorescence properties, bioimaging, and metal ion sensing aspects, have been extensively synthesized [12,13,14]. N-CDs can be derived in two main ways [15]: (1) employing small molecular precursors which possess higher nitrogen contents to synthesize N-CDs from bottom-up methods [16,17,18,19], as the obtained products usually have higher fluorescence quantum yield (QY) but involve a complicated process. (2) Using natural biomass precursors which contain a carbon and nitrogen source together to prepare N-CDs by top-down methods. In an especially green economical hydrothermal treatment [20,21,22], these N-CDs often possess relatively low QY. By contrast, natural biomass materials are ubiquitous, nontoxic, cheap, and reproducible. Thus, they are still growing challengeable precursors in the preparation of N-CDs.

Hg^2+^ is one of the most toxic and threatening heavy metal ions in the environment, can easily bioaccumulate in the human body and seriously damage the central nervous and endocrine systems [23,24,25]. Nowadays, complicated instruments and tedious procedures are obvious disadvantages of methods for Hg^2+^ detection [26,27,28]. Hence, it is still a huge task to develop an economical, environment-friendly, water-soluble, and fast method for the practical detection of trace Hg^2+^ in aqueous solutions. So far, studies have shown that CDs and N-CDs can be used as probes for the detection of Hg^2+^. The sensing mechanism is on account of FL quenching of CDs/N-CDs by Hg^2+^ speculatively due to the formation of nonradiative coordination compound between CDs/N-CDs and Hg^2+^. These CDs/N-CDs shows high selectivity and sensitivity towards Hg^2+^ [2,14,22,29].

Herein, we report on a green synthesis of N-CDs from natural biomass Highland barley for the detection of Hg^2+^. Highland barley is a unique cold and drought resistant crop grown in plateau regions. It is composed of 73.2% carbohydrate and 8.1% protein, and its price is very low (around 5 RMB/500 g in Qinghai province of China) so we choose it as a representative of Carbon-rich natural biomass to for the research. Taking into consideration that the N content is not enough high in Highland barley, ethanediamine is chosen as a nitrogen resource to prepare N-CDs. As expected, water-soluble N-CDs with QY 14.4% are obtained from Highland barley and ethanediamine via the green, low-cost and one-pot hydrothermal method. Further, Hg^2+^ can sensitively and selectively quench the fluorescence (FL) intensity of the as-synthesized N-CDs.

## 2. Materials and Methods

### 2.1. Materials and Reagents

Highland barley (Qinghai Academy of Agriculture and Forestr, Xining, China) was provided by Qinghai Academy of Agriculture and Forestry, washed and smashed before use. All chemicals were of analytical grade and were used as received. Ultrapure water was prepared by the Milli-Q Direct 16 device (Millipore, Boston, MA, USA).

### 2.2. Instruments

The UV/Vis spectra were recorded on a Shimadzu UV-2550 spectrophotometer (Shimadzu, Kyoto, Japan). The FTIR spectra were performed on a Perkin Elmer Spectrum Two™ spectrometer (Perkin Elmer, Waltham, MA, USA). The sample was diluted by KBr (Damao chemical reagent factory, Tianjin, China) and pressed into the disc. The Fluorescence measurements were acquired on a Cary Eclipse fluorescence spectrophotometer (Agilent Technologies, Santa Clara, CA, USA). The X-ray diffraction (XRD) patterns were carried out on a Rigaku D/Max 2500 PC X-ray diffractometer (Rigaku, Kyoto, Japan) in the range of 0° to 90° at a scanning speed of 4° min^−1^. The morphology and microstructure of the NCDs were verified through transmission electron microscopic (TEM) images and high-resolution TEM (HRTEM) images using a JEOL JEM-1200EX microscopy (JEOL (BEIJING) CO., LTD., Beijing, China.) with an accelerating voltage of 200 kV. The samples were prepared by dropping a water solution onto a 300-mesh copper grid deposited with a carbon film. The chemical composition of CDs was investigated by X-ray photoelectron spectroscopy (XPS) with a Thermo Scientific^TM^ ESCALABTM 250Xi X-ray Photoelectron Spectrometer (Thermo Scientific, Waltham, MA, USA).

### 2.3. Preparation of N-CDs

N-CDs were prepared by a hydrothermal method from Highland barley as carbon source and ethanediamine as nitrogen resource. In detail, 4.0 g Highland barley powder and 1.33 mL ethanediamine were added into 30 mL ultrapure water. Subsequently, the mixture was stirred and transferred into a 50 mL Teflon-lined autoclave (Xi’an Hong Chen Instrument Factory, Xi’an, China) and heated at 200 °C for 24 h in an oven. After cooling to room temperature, the product was centrifuged at 12,000 rpm for 20 min, then dialysed against deionized water through a dialysis membrane (MWCO = 1 kD) (Union Carbide Corporation, Texas City, TX, USA) for 24 h. Finally, the solution was dried under vacuum freeze-dryer (Anhui Zhongke Duling Commercial Electric Appliance co. LTD, Hefei, China) until a dark brown product was obtained.

### 2.4. Detection of Hg^2+^

Firstly, 3 mL N-CDs aqueous solution (0.05 mg mL^−1^) was added to a 1 cm cuvette, then 3 µL Hg^2+^ aqueous solution at different concentrations was injected into the above solution and mixed evenly, the FL intensities of the mixed solutions were measured at room temperature using a 400 nm excitation wavelength.

### 2.5. Investigation of Selectivity and Competitiveness

To investigate the selectivity of the N-CDs, nine kinds of other biological and environmental relevant metal ions (including Cu^2+^, Mg^2+^, Co^2+^, Zn^2+^, Mn^2+^, Cd^2+^, Ca^2+^, Pb^2+^, Ba^2+^, 1 mM) were respectively added to the solution of N-CDs (0.05 mg mL^−1^), and the FL intensity changes without and with these ions were recorded. Then, Hg^2+^ ions (0.5 mM) were injected into N-CDs solutions containing these metal ions. The FL intensities in the absence of Hg^2+^ ion were compared with the presence of Hg^2+^ ions.

## 3. Results and Discussion

### 3.1. Synthesis and Characterization of the N-CDs

**Figure 1:**
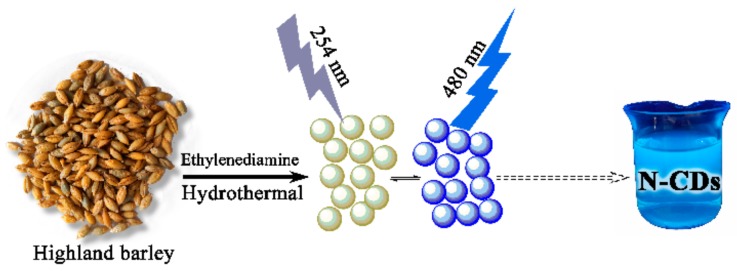
The preparation process of the N-CDs by hydrothermal method from Highland barley.

Highland barley contains plenty of carbohydrate and protein; the N-CDs were synthesized by a hydrothermal method at 200 °C for 24 h from Highland barley and ethanediamine as shown in Figure 1. When N-CDs were obtained, under the irradiation of UV light (254 nm) in an aqueous solution, very strong blue luminescence could be seen.

We investigated the influence of the precursor’s dosage on QY of the products. Firstly, the effect of the dosage of Highland barley (2, 4, 6, 8 g) was studied, the result was shown in Figure S1. When the dosage of Highland barley increased from 2 g to 4 g, the QY of CDs (without addition of ethanediamine) increased sharply and then slightly reduced when the mass was greater than 4 g. We speculated that the volume of autoclave was only 50 mL, too much mass would decrease the carbonization effect, so 4 g was chosen as the optimum quality of Highland barley. Subsequently, the influence of the mass of ethanediamine (0.33, 0.67, 1.33, 3.33 mL) was researched, and the result is shown in Figure S2. When the mass of ethanediamine increased from 0.33 mL to 1.33 mL, the QY of N-CDs largely increased then basically kept the same when mass of ethanediamine was more than 1.33 mL, hence we chosen 1.33 mL as the optimal mass of ethylenediamine.

**Figure 2:**
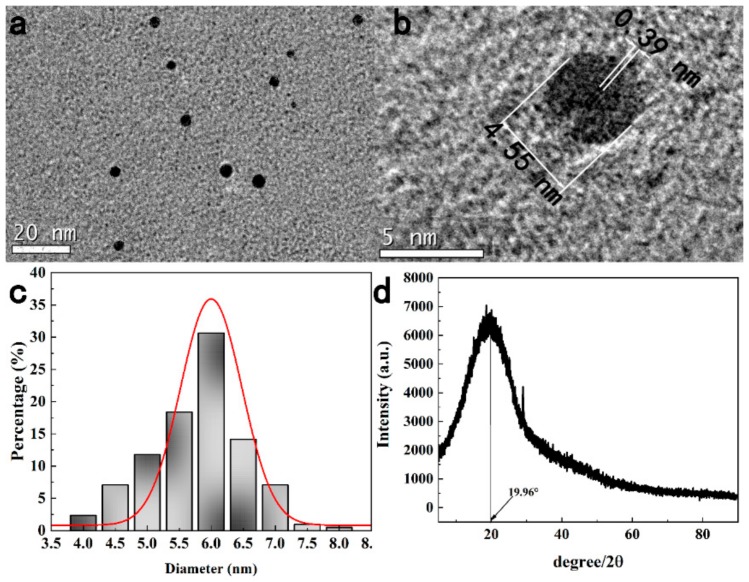
(**a**) TEM image of N-CDs, ratio scale is 20 nm. (**b**) HRTEM image of N-CDs, ratio scale is 5 nm. (**c**) Diameter distribution of N-CDs. (**d**) XRD pattern of N-CDs.

The formation of N-CDs was confirmed by TEM as shown in Figure 2a. The N-CDs were nearly quasi-spherical in shape and mono-dispersion. The HRTEM image in Figure 2b shows that the lattice spacing of ca. 0.39 nm corresponded to that of a graphitic amorphous structure. The particle size distribution histogram in Figure 2c demonstrates that their diameters were very small and mainly distributed in the range from 4.5–7 nm and the average diameter was 5.8 nm. The XRD pattern of the N-CDs in Figure 2d displayed a broad reflection peak at 2θ = 19.96°, and determined that the interlayer spacing *d* was 0.44 nm, which became larger than graphite (0.34 nm). The increase of the *d* value showed an increase of the amorphous nature which can attributed to more defect sites being introduced to the product through increased N-doping to the crystal lattice [30,31].

**Figure 3:**
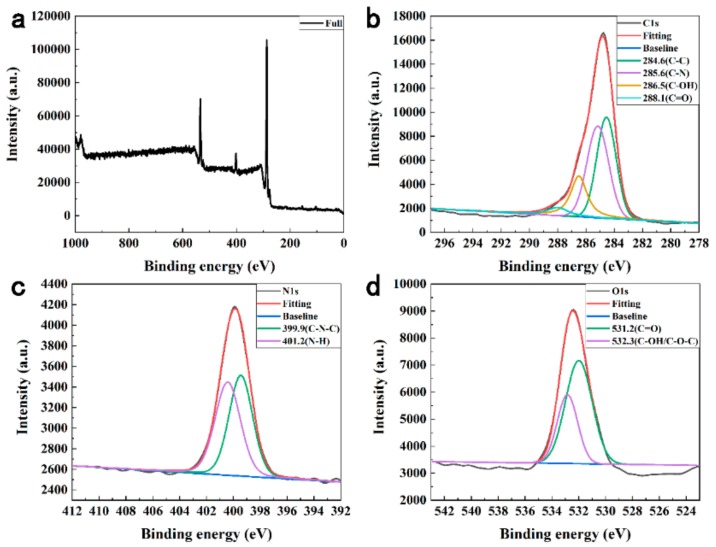
(**a**) XPS full spectrum of N-CDs. (**b**)–(**d**): High-resolution C1s, O1s and N1s peaks of the N-CDs, respectively.

The surface functional groups and element analysis of N-CDs were confirmed by XPS. As shown in Figure 3a, N-CDs displayed three predominant strong peaks at 284.7, 399.9, and 532.4 eV, which were ascribed to C1s, N1s and O1s peak, respectively [32,33,34]. The ratio of C:N:O was 80.75: 5.45:13.80. The high-resolution C1s spectrum was shown in Figure 3b, according to the previous studies, the spectrum could be deconvoluted into four peaks at around 284.6, 285.7, 286.8, and 288.1 eV, which were attributed to C–C, C–N, C–OH, C=O groups [35,36], severally. In the high-resolution N1s spectrum shown in Figure 3c, the two peaks at 399.4 and 400.5 eV could be assigned to C–N–C and N–H groups [37,38,39], respectively. From the O1s spectrum shown in Figure 3d, the two fitted peaks at 532.0 and 532.8 eV were ascribed to C=O and C–OH/C–O–C groups [40,41], severally.

**Figure 4:**
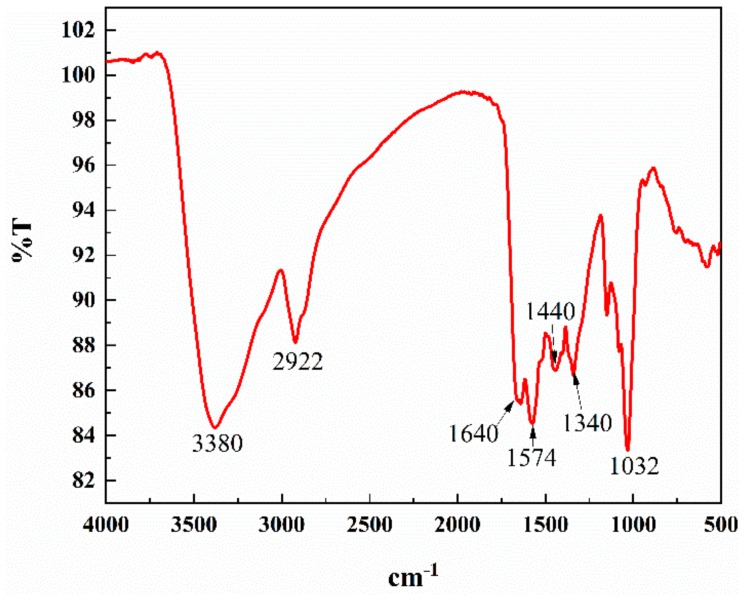
The FTIR spectrum of the N-CDs.

The FTIR spectrum also was used to verify the functional groups of the synthetic N-CDs in Figure 4. The characteristic absorption band at 3380 cm^−1^ was corresponding to the stretching vibrations of O–H and N–H bonds. The peak at 2922 cm^−1^ was ascribed to the C–H bonds. The absorption bands at 1640 and 1440 cm^−1^ were due to the COO^−^ stretching vibrations. The characteristic absorption band of C–N at 1574 cm^−1^ was also observed. The band centered at 1340 cm^−1^ was assigned to the vibrations of the C–H bond. Furthermore, the peak at 1032 cm^−1^ can be attributed to the C–O band [2,42,43].

It could be confirmed from the conclusive evidence of FTIR and XPS data that the as-synthesized N-CDs owned plenty of hydrophilic groups, such as hydroxyl (–OH), carboxylic (–COOH), and amidogen (–NH_2_) moieties. These groups were helpful to the enhancement of aqueous solubility of N-CDs for further sensing application in water solution.

### 3.2. Optical Properties of the N-CDs

**Figure 5:**
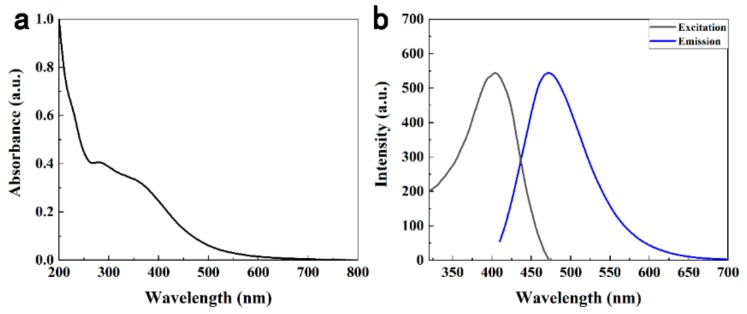
(**a**) UV-vis absorbance and (**b**) FL spectra of N-CDs.

To explore the optical properties of the N-CDs, UV-vis absorption and FL spectra were studied at room temperature. In Figure 5a, N-CDs showed a inapparent absorption peak at about 280 nm [44], which was ascribed to the n-π* transition of C=O bond [4]. Figure 5b displayed the excitation and emission traits of the N-CDs, under excitation at 400 nm the strongest emission peak centered at 480 nm appeared, indicating the N-CDs with fluorescence. The quantum yield (QY) of the N-CDs was about 14.4% compared with quinine sulphate [34], and the higher QY was possible owing to nitrogen-containing functional groups which could validly passivate the surface active sites of the carbon dots. Thus the FL properties of the N-CDs were increased [41], and because of that the QY of our N-CDs was higher than most of N-CDs from biomass [11,20,21,22].

**Figure 6:**
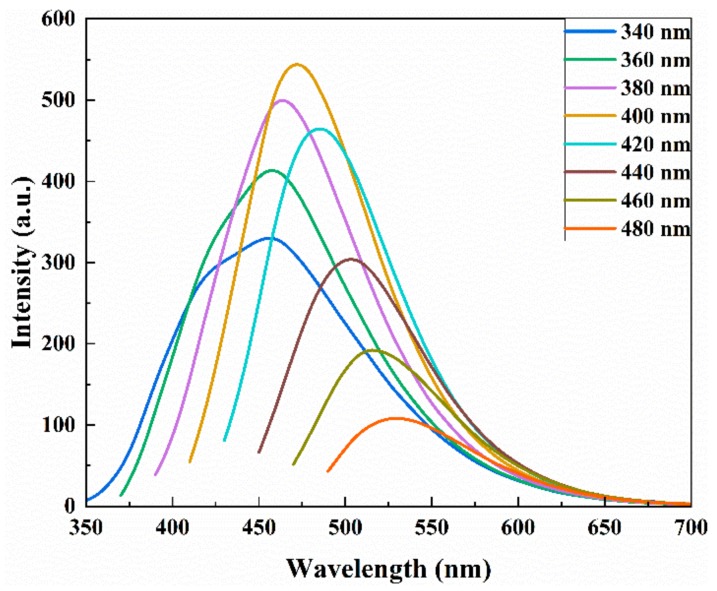
Excitation wavelength-dependent FL emission spectra of the N-CDs.

To further investigate the optical properties, the FL emission spectra of the N-CDs were recorded at various excitation wavelengths. As shown in Figure 6, the FL spectra presented a representative excitation wavelength-dependent characteristic. With the increase of the excitation wavelength from 340 to 400 nm by an increment of 20 nm, the FL intensities gradually enhanced. The strongest emission peak appeared at the excitation wavelength of 400 nm. When the excitation wavelength changed from 400 nm to 480 nm, the FL emission peaks red-shifted along with the FL intensities and decreased [45]. These behaviors were perhaps due to the different sizes of N-CDs (Figure 2c) and different surface emission sites which came from abundant organic groups (–OH, –COOH and –NH_2_) on the N-CDs’ surfaces [46].

To research luminescent mechanism of the N-CDs, a time-correlated single-photon counting technique was used to record the FL decay profile of the N-CDs excited at 360 nm (Figure S5). The data of FL lifetime were well fitted to a triple-exponential function, the mean FL lifetime of 5.34 ns was calculated for the N-CDs. The fast component (*τ*_1_ ~1.18 ns) has the amplitude of 15.28%, while the *τ*_2_ (~3.81 ns) and *τ*_3_ (~10.79 ns) component are around 60.74% and 23.98%, respectively. The FL lifetime is similar to those as-reported [47,48]. Such a short lifetime indicated that luminescent mechanism of the N-CDs could be the radioactive recombination of excitations [10]. The stability of the N-CDs on different test conditions was studied, the results were shown in Figures S3 and S4. The FL intensity of N-CDs under different pH values from 2 to 10 was measured (Figure S3), in general, pH values did not generate huge change among pH 2–10, Figure S4 showed that the FL intensity varied only slightly with the temperature changing from 10 °C to 50 °C. The results show that the prepared N-CDs have good stability, making them hopeful for future applications.

### 3.3. Fluorescence Detection of Hg^2+^

**Figure 7:**
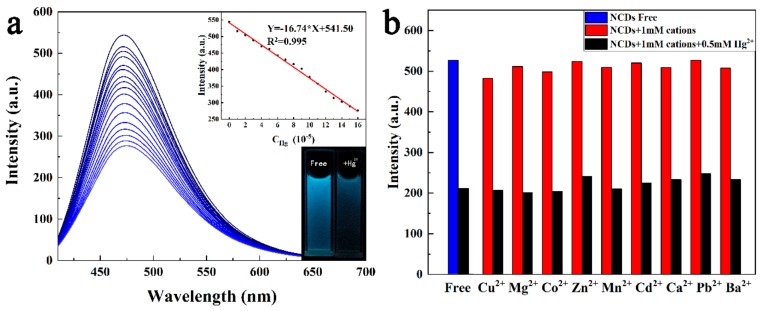
(**a**) FL emission spectra of the N-CDs (0.05 mg mL^−1^) upon exposure to various concentrations of Hg^2+^ (from top to bottom: 0, 10, 20, 30, 40, 50, 60, 70, 80, 90, 100, 110, 120, 130, 140, 150, 160 μM) in aqueous solution excitation at 400 nm; Inset 1 (bottom right corner) shows the fluorescence images of the N-CDs in aqueous solution in the absence and presence of Hg^2+^ ion under UV light of 254 nm. Inset 2 (top right corner) shows the calibration curves and linear equation for FL intensity and concentration of Hg^2+^. (**b**) The selectivity test of N-CDs (0.05 mg mL^−1^) for Hg^2+^ against different metal ions.

The N-CDs possess a possible ability to be used to detect metal ions in that the surface of N-CDs attached plenty of oxygen functional groups, as these groups coordinate with metal ions, the FL intensity of N-CDs will decrease obviously [49]. We investigated the Hg^2+^ response to the N-CDs, with a gradual addition of Hg^2+^ to the N-CDs aqueous solution. The FL intensity (in 480 nm) of the N-CDs decreased regularly as shown in Figure 7a, indicating that Hg^2+^ ion could sensitively quench the FL of the N-CDs. The highly efficient quenching of the N-CDs′ FL is likely due to the strong chelating ability of Hg^2+^ toward the carboxylic group on N-CDs surface, resulting in the formation of non-fluorescent complex and facilitating non-radiative electron/hole recombination annihilation by an effective electron transfer process, causing the static quenching of the FL of N-CDs [50,51]. The curve inserted in Figure 7a shows the relationship between the FL intensity of the N-CDs and Hg^2+^ ion concentration. In the range of 10–160 μM the linear equation of FL intensity versus the concentration was FL = −16.74 × C_Hg_^2+^ + 541.5, R^2^ = 0.995. According to three times the standard deviation regulation [25], we figured out that the limit of detection (LOD) was equal to 0.48 μM, which was higher than the safety level stipulated by the EPA (0.01 μM) for drinking water. We compared the properties of our N-CDs with some reported CDs for Hg^2+^ as shown in Table 1. Although the LOD of our N-CDs was not low enough, for biomass as precursor of CDs, the as-prepared N-CDs owned higher QY and wider linear range, thus it was possibly used to detect Hg^2+^ in samples where regulations for Hg^2+^ ion are less severe. For example, it can test Hg^2+^ in drugs, biological products and so on [48]. 

The selectivity of the N-CDs for different metal ions was also investigated in this work. We chose nine kinds of other biological and environmental relevant metal ions including Cu^2+^, Mg^2+^, Co^2+^, Zn^2+^, Mn^2+^, Cd^2+^, Ca^2+^, Pb^2+^, Ba^2+^. When these ions were respectively added in the solution of N-CDs, no obvious FL intensity change occurred, as shown in Figure 7b. But, after injecting Hg^2+^ into N-CDs solution containing other metal ions, Hg^2+^ could validly quench the FL of N-CDs solution. This result proved the highly selectivity of N-CDs for Hg^2+^ over other competitive metal ions.

## 4. Conclusions

In summary, we synthesized completely water-soluble N-CDs by a green hydrothermal method at 200 °C for 24 h from biomass using Highland barley as a carbon source and ethanediamine as nitrogen source. Due to the formation of nitrogen-containing functional groups from ethanediamine, the QY of N-CDs was as high as 14.4%. The FL spectra of the N-CDs presented a representative excitation wavelength-dependent characteristic. It was found that Hg^2+^ ions could be sensitively and selectively detected by efficiently quenching the FL of the N-CDs. In addition, Highland barley constitutes an abundant and cheap form of biomass, and it is expected to be used to prepare N-CDs on a large scale. 

## Figures and Tables

**Table 1:** Comparison of different CDs for Hg^2+^.

FL Probe	Precursor	QY (%)	LOD (μM)	LinearR (nM)	Ref.
CPs	Pomelo peel	6.9	0.00023	0.5–10 and 500–4 × 10^4^	2
N-CQD	folic acid	15.7	0.23	0–2.5 × 10^4^	14
FNCPs	strawberry juice	6.88	0.003	10–5 × 10^4^	22
N,S/C-dots	Citric acid/urea/L-cysteine	25.2	2	0–4 × 10^4^	29
N-CDs	Highland barley/ethanediamine	14.4	0.48	1× 10^3^–16 × 10^4^	This work

## Supplementary Materials

The following are available online at https://www.mdpi.com/1424-8220/19/14/3169/s1. Figure S1. The influence of the Highland barley (HB) dosage on QY of the products, Figure S2. The influence of the ethanediamine (EDA) dosage on QY of the N-CDs, Figure S3. The influence of the pH values from 2 to 10 on the FL intensity of the N-CDs, Figure S4. The influence of the temperature from 10 °C to 50 °C on the FL intensity of the N-CDs, Figure S5. FL decay spectrum of the N-CDs at 458 nm (excitation at 360 nm).
