# Supplementary material for: Green Hydrothermal Synthesis of N-doped Carbon Dots from Biomass Highland Barley for the Detection of Hg2+
Source: Sensors (Basel). 2019 Jul 18;19(14):3169. doi: 10.3390/s19143169 (PMC6679557; doi:10.3390/s19143169)
Supplement: Supplementary file 1 [file sensors-19-03169-s001.pdf]

Supplementary data

Green hydrothermal synthesis of N-doped Carbon dots from biomass Highland barley for the detection of Hg^{2+}

Yadian Xie [a], Dandan Cheng [b], Xingliang Liu [a,*], Aixia Han [a,*]

[a]Chemical Engineering College, Qinghai University, Xining 810016, Qinghai, China;.

[b]School of Life Science, Wuchang University of Technology, Wuhan 430223, Hubei, China.

*Corresponding authors:

Xingliang Liu: liuxl1219@163.com; Aixia Han: hanaixia@tsinghua.org.cn.

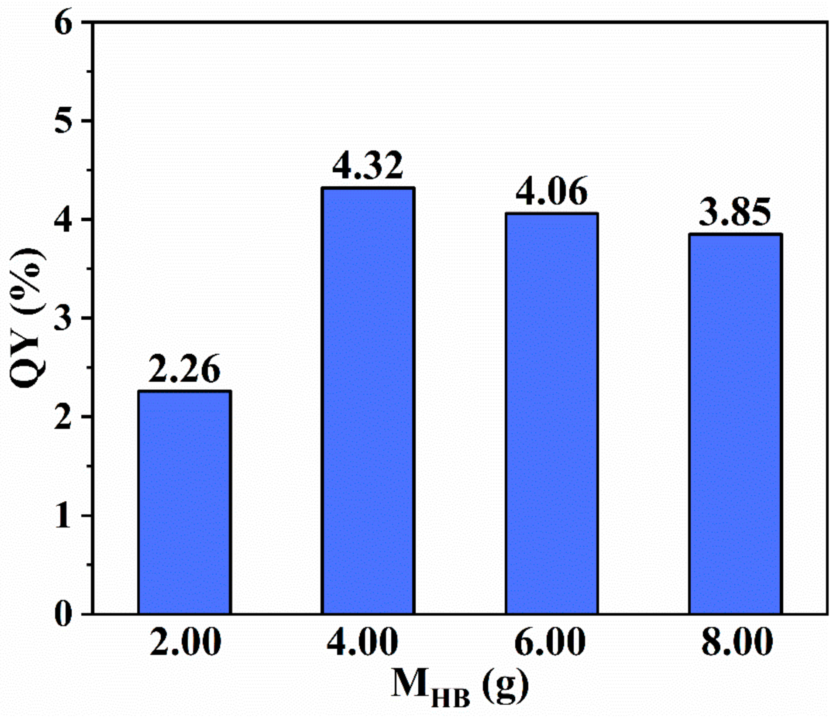

Figure S1. The influence of the Highland barley (HB) dosage on QY of the products.

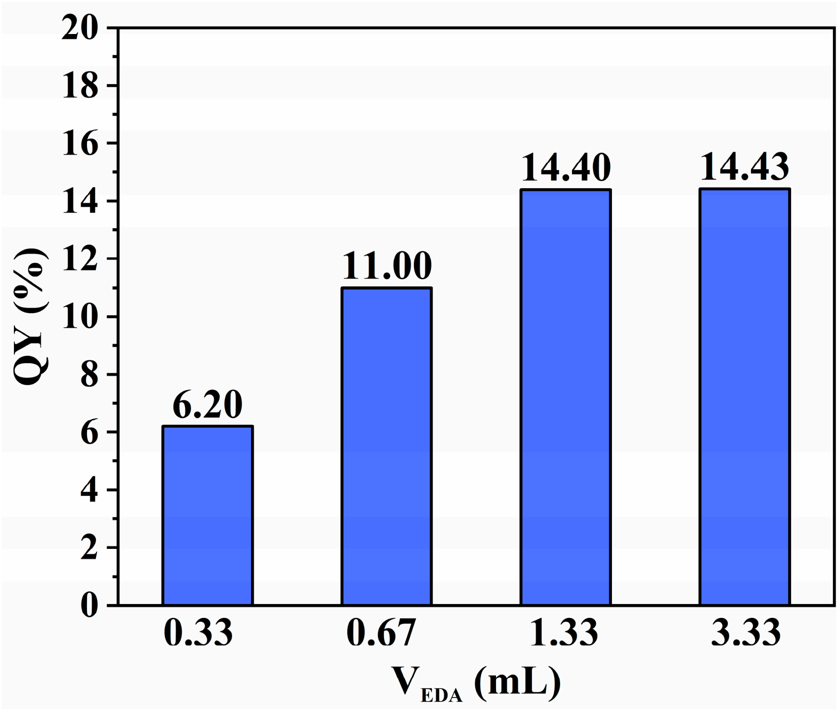

Figure S2. The influence of the ethanediamine (EDA) dosage on QY of the N-CDs.

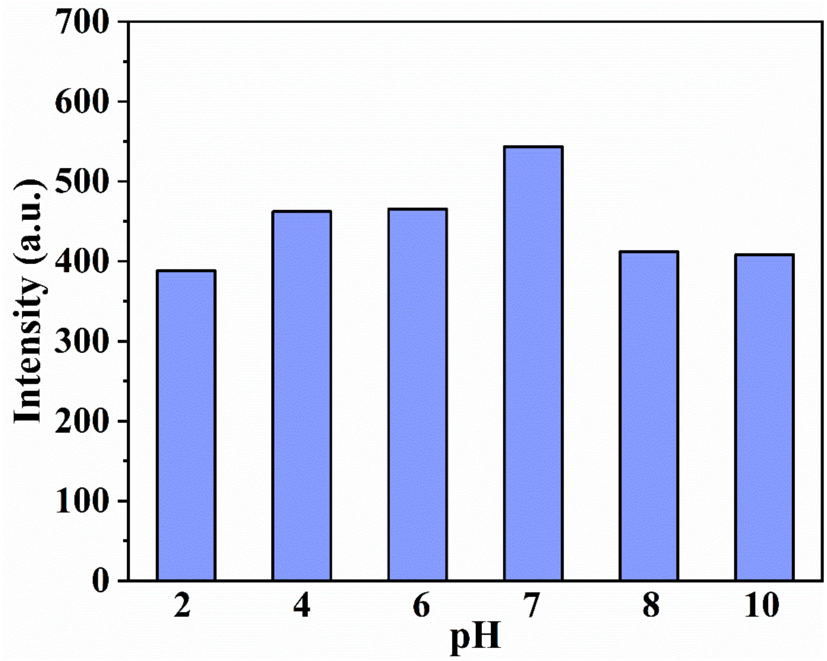

Figure S3. The influence of the pH values from 2 to 10 on the FL intensity of the N-CDs.

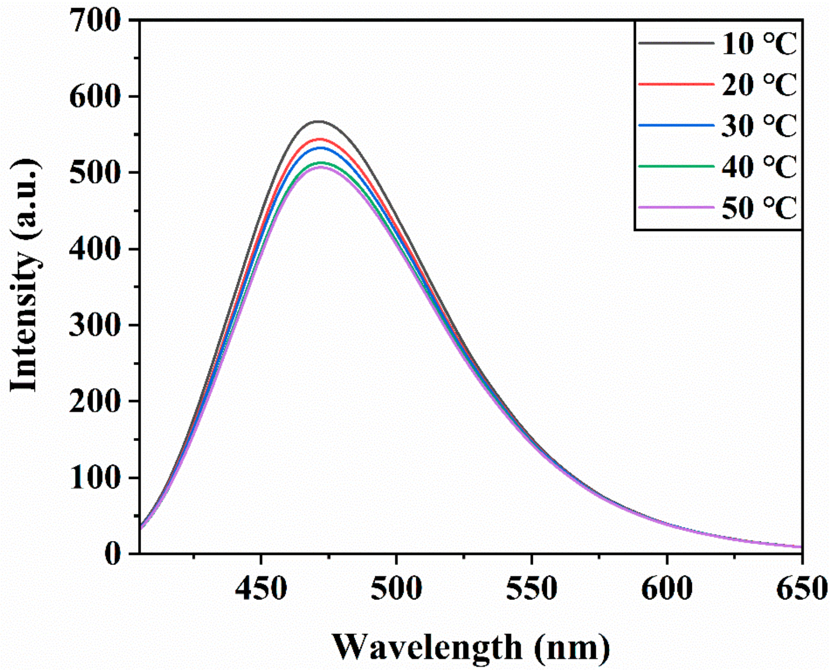

Figure S4. The influence of the temperature from 10 ^{O}C to 50 ^{O}C on the FL intensity of the N-CDs.

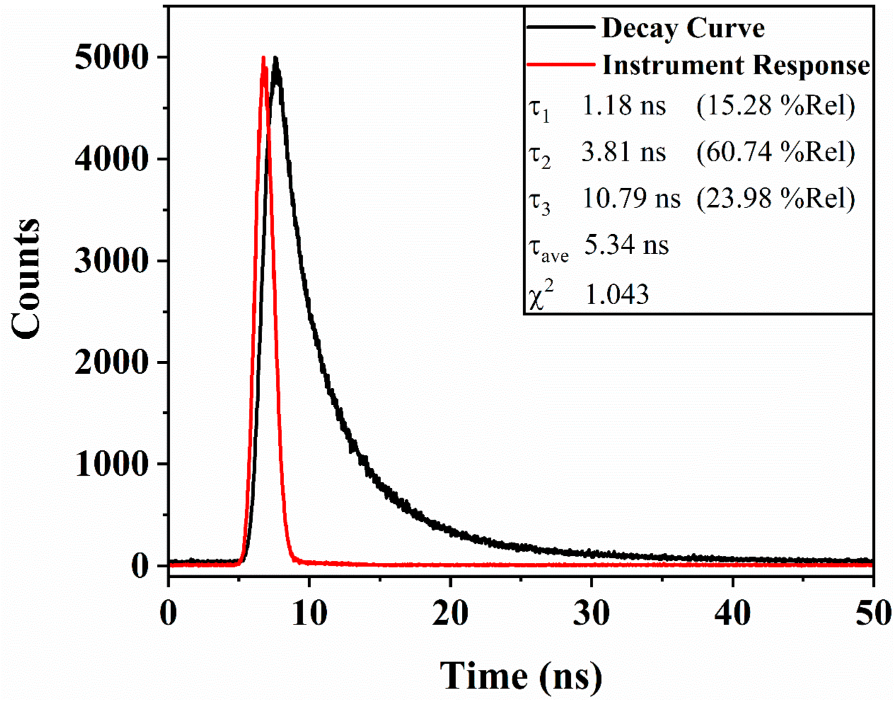

Figure S5. FL decay spectrum of the N-CDs at 458 nm (excitation at 360 nm).
